# A Case of Myasthenia Gravis: A Paraneoplastic Syndrome or an Immune-Related Disorder?

**DOI:** 10.7759/cureus.78640

**Published:** 2025-02-06

**Authors:** Jaya S Thyagarajan, Mohammad H Almomani, Nithisha Thatikonda, Chilvana Patel, Xiang Fang

**Affiliations:** 1 Medicine, John Sealy School of Medicine, University of Texas Medical Branch, Galveston, USA; 2 Neurology, University of Texas Medical Branch, Galveston, USA

**Keywords:** anti-titin antibody, immune check point inhibitors (icis), immune-related adverse effects, immune-related myasthenia gravis, immunotherapy-related adverse events, myasthenia gravis, renal cell carcinoma, steroid refractory, striated muscle antibodies

## Abstract

Myasthenia gravis (MG) is an autoimmune neuromuscular disease characterized by fatigable muscle weakness. While commonly linked to acetylcholine receptor (AChR) antibodies, other reported antibodies include muscle-specific kinase (MuSK), low-density lipoprotein receptor-related protein 4 (LRP4), agrin, striated muscle, myosin, ryanodine receptor, and titin. Notably, titin antibodies are being highlighted for their role in MG pathogenesis, as they have been associated with increased disease severity. Immune checkpoint inhibitors (ICIs), while highly effective for solid tumors, can rarely induce immune-related myasthenia gravis (irMG), a neurological adverse effect with higher mortality than classic MG. We present a case of MG with atypical serology, highlighting the challenges of classifying and treating classic MG, paraneoplastic syndromes, and irMG.

A 68-year-old man with a history of stage IV left renal cell carcinoma (RCC) with lung metastases after undergoing left nephrectomy and right segmental lung lobectomy presented with one month of palpebral ptosis, shortness of breath, dysphagia, and generalized muscle weakness. He had recently received two doses of 200 mg pembrolizumab, a programmed cell death protein 1 (PD-1) inhibitor. Physical examination revealed bilateral palpebral ptosis, symmetric, primarily proximal weakness, including bulbar and facial muscles with fatigability, and normal reflexes. Serum studies were positive for anti-striated muscle (>1:2560) and anti-titin (>2.71) antibodies, suggesting irMG or a paraneoplastic syndrome, with negative anti-AChR and anti-MuSK antibodies. Computed tomography (CT) of the chest showed no thymic disease. The patient improved with high-dose steroids followed by plasma exchange and intravenous immunoglobulin (IVIG). Repeated striated muscle antibodies were decreased, but anti-titin antibodies were persistently elevated. He is neurologically stable on pyridostigmine 60 mg, 3 liters of nasal cannula oxygen, and nighttime bilevel positive airway pressure (BiPAP) but experiences residual muscle weakness that requires wheelchair use.

In summary, we present a case of MG with the presence of titin and striated muscle antibodies in a patient with metastatic RCC undergoing checkpoint immunomodulating therapy. Management remains challenging in patients with underlying malignancy treated with ICIs.

## Introduction

Myasthenia gravis (MG) is a neuromuscular junction disease characterized by fatigable generalized muscle weakness, with a worldwide prevalence of 2.19 to 36.71 cases per 100,000 [1]. While MG is frequently associated with thymomas (10% to 20%), it has been reported sporadically in malignancies such as lung cancer and renal cell carcinoma (RCC) [2-4]. Myasthenia gravis has also been reported as a rare but life-threatening outcome of immune checkpoint inhibitor (ICI) therapy [5]. Immune checkpoint inhibitors, which block inhibitory T-cell signals that are otherwise exploited by cancer cells to promote tumor growth, have become a cornerstone in oncological treatment over the past few decades [6,7]. These therapies target molecular pathways such as programmed cell death protein 1 (PD-1) (e.g., pembrolizumab), PD-1 ligand (e.g., atezolizumab), and cytotoxic T-lymphocyte-associated protein-4 (CTLA-4) (e.g., tremelimumab) [6,7]. However, by modifying the physiological T-cell response, ICIs can trigger autoimmune adverse effects such as immune-related myasthenia gravis (irMG) [5]. As ICIs are fundamental for the treatment of solid malignancies, it is crucial to distinguish whether new-onset MG arises as an immune-related adverse effect or as a paraneoplastic phenomenon of the primary tumor. This distinction is essential for guiding the management of these patients.

Identifying the specific antibodies present in irMG and paraneoplastic MG, as opposed to classic MG, may assist in making this separation. In classic MG, antibodies against acetylcholine receptors (AChR) are the most common (~85%), followed by antibodies against muscle-specific kinase (MuSK) and low-density lipoprotein receptor-related protein 4 (LRP4) (both < 10%) [1,8]. Studies have shown a high prevalence of AChR antibodies in both irMG and RCC-associated MG [1,3,5,8]. Emerging antibodies of interest for patients who are negative for anti-AChR include antibodies against striated muscle, such as those targeting titin, myosin, and the ryanodine receptor [8]. Specifically, anti-titin antibodies have been identified as a sensitive marker of severe MG and thymomas [4,9,10]. Titin is also being highlighted as a key marker in irMG, but this relationship remains poorly understood, with fewer than a dozen reported cases in the literature [11]. Similar to thymoma-associated MG, the presence of titin antibodies in irMG may predict worse outcomes and higher fatality [11,12]. Therefore, early detection and aggressive treatment of anti-titin irMG may be necessary to achieve remission and reduce mortality.

We present a case of a patient with metastatic RCC treated with pembrolizumab, a PD-1 inhibitor, who developed MG with anti-striated muscle and anti-titin antibodies. This report underscores the complex, overlapping associations between thymoma-associated MG, paraneoplastic associations, and immune dysregulation resulting from ICI therapy.

## Case presentation

The patient was a 68-year-old man with a medical history of hypothyroidism and stage IV metastatic left RCC who had previously received nephrectomy and segmental lobectomy. He had recently received two sessions of intravenous pembrolizumab 200 mg for metastatic disease. Twenty-one days after his second infusion, he developed diarrhea. Laboratory findings revealed mildly elevated liver enzymes. This transaminitis was persistent when measured two weeks later. Additionally, the patient developed episodic, fatigable right palpebral ptosis and generalized weakness that worsened at night, prompting the initiation of prednisone 100 mg daily for two weeks. Following steroid therapy, his liver function tests had improved but did not return to baseline. Due to the persistence of ptosis, serological tests were ordered for AChR binding and blocking, striated muscle, MuSK, and titin antibodies. One month later (90 days after the first infusion and 70 days after the second infusion), the patient was admitted due to worsening bilateral ptosis, shortness of breath, diffuse muscle fatigability, dysphagia, and weight loss.

Upon admission, physical exam findings showed bilateral ptosis (but intact other cranial nerves) and diffuse muscle weakness with fatigability. Muscle weakness was symmetric and more pronounced in the neck and proximal axial muscles (3+). There was bulbar and facial muscle involvement, including dysphagia and dysphonia. Deep tendon reflexes were normal. The initial work-up showed slightly elevated alanine aminotransferase (ALT), mildly elevated creatine kinase (CK), elevated lactate dehydrogenase (LDH), elevated blood urea nitrogen (BUN), elevated creatinine, elevated thyroid-stimulating hormone (TSH), and elevated total and unconjugated bilirubin. Free triiodothyronine (T3), free thyroxine (T4), aspartate aminotransferase (AST), alkaline phosphatase (ALP), and troponin were within normal limits (Table 1).

**Table 1:** Laboratory Findings on Admission BUN: Blood Urea Nitrogen; TSH: Thyroid-Stimulating Hormone; T3: Triiodothyronine; T4: Thyroxine; AST: Aspartate Aminotransferase; ALT: Alanine Aminotransferase; ALP: Alkaline Phosphatase; ESR: Erythrocyte Sedimentation Rate; CRP: C-reactive Protein

Laboratory Value	Hospital Day 1	Reference Range
Platelet count	↓79	150 – 400
BUN	↑37	7 – 24
Creatinine	↑2.57	0.6 - 1.2
Unconjugated Bilirubin	↑1.3	< 1.2
Total Bilirubin	↑1.5	< 1.2
Creatinine Kinase	↑253	40 – 150
Lactate Dehydrogenase	↑294	135 – 225
Troponin	0.027	< 0.04
TSH	↑15.40	0.27 – 4.2
Free T3	3.59	2.3 – 4.1
Free T4	1.64	0.9 – 1.7
AST	37	< 35
ALT	↑51	< 45
ALP	51	30–120
ESR	2	< 20
CRP	0.4	< 0.9

Extensive serological tests conducted prior to admission revealed high titers of anti-titin and anti-striated muscle antibodies, while AChR and MuSK antibodies were negative (Table 2). Paraneoplastic antibodies (neuronal/amphiphysin, Purkinje cell, crossveinless 2.1 (CV2.1), and SRY-box transcription factor 1 (SOX1) antibodies) were also negative. Electromyography (EMG) was deferred as serological tests had already confirmed myasthenia gravis. The presence of anti-titin antibodies raised concerns about thymoma or thymic hyperplasia associated with MG, but a computed tomography (CT) scan of the thorax ruled them out. Immune-regulated overlap syndromes such as myositis and myocarditis were ruled out by normal troponin levels, a normal left ventricular ejection fraction on echocardiogram, the absence of myalgias, and relatively normal CK and erythrocyte sedimentation rate (ESR) levels.

**Table 2:** Neuromuscular Antibody Findings

Laboratory Value	24 Days Prior to Admission	Hospital Day 20	Reference Range
Anti-nuclear Antibody	1:320	–	< 1:40
Acetylcholine-Binding Antibody	0.2	0.4	< 0.4
Muscle-Specific Kinase Antibody	< 1:10	–	< 1:10
Anti-striated Muscle Antibody	> 1:2560	1:40	< 1:40
Anti-titin Antibody	> 2.71 IV	> 2.71 IV	0 – 0.4

Treatment and outcomes

The patient was closely monitored for respiratory compromise upon admission, with an initial vital capacity of 2 liters and a negative inspiratory force of -40 cm water (H₂O). Pyridostigmine 60 mg daily was initiated for myasthenic crisis and titrated up to 120 mg three times daily. Starting on hospital day two, the patient received five sessions of plasmapheresis (PLEX) and five days of intravenous methylprednisolone 1 g, which was later transitioned to a tapering dose of 1.5 mg/kg/day prednisone. The patient reported symptomatic improvement in his muscle fatigability but was transferred to the ICU on the day after completing PLEX due to worsening dyspnea and a vital capacity of 1.1 liters. Pyridostigmine was held to avoid exacerbating airway secretions. In the ICU, the patient received five days of intravenous immunoglobulin (IVIG) at a dose of 2 g/kg and continued the steroid taper. Following IVIG treatment, the patient’s gait and strength improved, with muscle strength returning to 5/5 in all muscles except for the deltoids, biceps, and hip flexors (4+). The patient was able to ambulate within his room and stand for minutes at a time, and his ptosis and voice hoarseness were persistent. He was transferred out of the ICU and restarted on pyridostigmine. Repeat testing showed a significant decrease in striated muscle antibody titers (1:40), though the titin antibody titer remained elevated (>2.71 IV).

After discharge, the patient received two maintenance sessions of IVIG at a dose of 1 g/kg over two days, administered every three weeks. Unfortunately, further IVIG treatment was discontinued due to the development of a pulmonary embolism, so he started maintenance mycophenolate 500 mg twice daily. The patient continues to experience residual muscle weakness, ptosis, and dysphonia but remains neurologically stable on pyridostigmine 60 mg, 3 liters of nasal cannula oxygen, and nighttime bilevel positive airway pressure.

## Discussion

Here, we present a patient with metastatic RCC who developed MG after ICI therapy, with positive titin and striated muscle antibodies. While these antibodies have been infrequently reported in irMG, emerging evidence suggests that titin antibodies may serve as biomarkers for predicting immune-related adverse effects (irAEs), including irMG in the absence of an underlying thymoma [11,13,14]. The persistently elevated titin antibody titers in this patient despite treatment likely suggest a refractory course of disease, consistent with his responses to various treatments.

Notably, a high prevalence (67% to 83%) of AChR antibodies has been documented in irMG patients [5,14,15]. However, our patient was negative for AChR antibodies both before and after MG treatment. In such cases, the absence of AChR antibodies should prompt consideration of alternative serological markers, such as anti-titin, anti-MuSK, anti-striated muscle, anti-ryanodine receptor (RyR), anti-low-density lipoprotein receptor-related protein 4 (anti-LRP4), and anti-agrin antibodies. It is also important to recognize that seronegative MG has been reported, highlighting the complexity of relying solely on serology in suspected irMG cases [16]. Early detection and prompt treatment with immunosuppressants, followed by IVIG or PLEX, are crucial to prevent progression to respiratory crisis and potential mortality [16].

While paraneoplastic syndromes occur in approximately one-third of both clear-cell and non-clear-cell RCC patients, few studies have investigated MG as an RCC-related syndrome [17]. A study of 283 MG patients reported a 2% incidence of RCC, which is significantly higher than the general population (<0.06%) [3]. Within this study, among the six patients with MG and RCC who had never used an ICI, all were anti-AChR positive, two had anti-titin (33%), two had anti-RyR (33%), and none had MuSK antibodies [3]. However, the majority of studies on MG in the presence of RCC occur after the initiation of ICIs, pointing to immune dysregulation as the likely precipitating event [16,18,19]. Whether RCC alone is a risk factor for the development of MG remains a mystery. Future reports of RCC-associated MG in the absence of ICI use would enable the comparison of autoantibodies between paraneoplastic and irMG, including whether anti-titin could be a useful diagnostic marker.

The delayed treatment timeline may have contributed to the progression of a life-threatening disease, which earlier intervention could potentially have prevented. The patient began experiencing symptoms of ptosis and fatigable muscle weakness within 32 days of his second pembrolizumab infusion, consistent with reports that irMG often manifests within the first three doses of ICI treatment [11]. However, there were delays in detecting and treating his condition. His initial symptoms of diarrhea and unilateral ptosis did not immediately raise suspicion for MG. Instead, he was placed on a two-week course of high-dose steroids for a suspected irAE, while testing for MG antibodies was delayed until the following month. By then, the patient had developed steroid-refractory irMG, requiring more aggressive treatments such as PLEX and IVIG.

Recent algorithms for managing immune-related neuromuscular toxicity based on the Common Terminology Criteria for Adverse Events (CTCAE) grading emphasize the importance of early intervention [20]. Current recommendations propose PLEX and IVIG as first-line treatments for grade 3 or 4 irMG [20]. For this patient, MG testing at the onset of ptosis could have enabled prompt initiation of PLEX and IVIG, which might have averted the patient's life-threatening respiratory distress. Additional treatment guidelines for irMG include long-term maintenance with oral steroids and symptomatic management with pyridostigmine [20]. Given the rising use of ICIs, it is increasingly important for clinicians to be vigilant and adhere to protocols for the close monitoring of irAEs.

## Conclusions

This case underscores the diagnostic and therapeutic challenges of managing MG in the context of ICI therapy, particularly with atypical antibody profiles. The presence of anti-titin and anti-striated muscle antibodies in a patient with metastatic RCC highlights the overlapping features of irMG and paraneoplastic syndromes. We are presenting all available data, which is a limitation of this study, as the atypical serology makes it difficult to definitively classify the etiology of MG in this patient. This report emphasizes the importance of early detection, as delayed diagnosis for this patient led to disease progression requiring intensive therapies. Despite initial improvement, the persistently elevated titin antibody titer suggests a refractory disease course. Current guidelines stress early intervention with PLEX or IVIG for severe irMG as well as the need for long-term immunosuppressive therapy. Clinicians must maintain a high index of suspicion for irMG in patients receiving ICIs and promptly address early disease manifestations to mitigate progression to life-threatening complications. Further research into the role of anti-titin antibodies as biomarkers and the relationship between RCC, MG, and ICIs is essential to improve outcomes for this complex demographic of patients.
